# Prognostic factors in primary diffuse large B-cell lymphoma of adrenal gland treated with rituximab-CHOP chemotherapy from the Consortium for Improving Survival of Lymphoma (CISL)

**DOI:** 10.1186/1756-8722-5-49

**Published:** 2012-08-13

**Authors:** Yu Ri Kim, Jin Seok Kim, Yoo Hong Min, Dok Hyun Yoon, Ho-Jin Shin, Yeung-Chul Mun, Yong Park, Young Rok Do, Seong Hyun Jeong, Joon Seong Park, Sung Yong Oh, Suee Lee, Eun Kyung Park, Joung-Soon Jang, Won-Sik Lee, Hwe-Won Lee, HyeonSeok Eom, Jae-sook Ahn, Jae-Heon Jeong, Sun Kyung Baek, Seok Jin Kim, Won Seog Kim, Cheolwon Suh

**Affiliations:** 1 Department of Internal Medicine, Yonsei University College of Medicine, Seoul, Korea; 2 Asan Medical Center, University of Ulsan College of Medicine, Seoul, Korea; 3 Pusan National University Hospital, Busan, Korea; 4 Ewha Womans University School of Medicine, Seoul, Korea; 5 Korea University College of Medicine, Seoul, Korea; 6 Keimyung University School of Medicine, Daegu, Korea; 7 Ajou University School of Medicine, Suwon, Korea; 8 Dong-A University College of Medicine, Busan, Korea; 9 Chung-Ang University Hospital, Seoul, Korea; 10 Busan Paik Hospital, Inje University College of Medicine, Busan, Korea; 11 National Cancer Center, Goyang, Korea; 12 Chonnam National University Hwasun Hospital, Hwasun, Jeollanamdo, Korea; 13 Kyung Hee University Medical Center, Seoul, Korea; 14 Samsung Medical Center, Sungkyunkwan University School of Medicine, Seoul, Korea; 15 Department of Internal Medicine, Division of Hematology, Yonsei University College of Medicine, 50 Yonsei-ro, Seodaemun-gu, Seoul, 120-752, Korea

**Keywords:** Primary adrenal lymphoma, Diffuse large B-cell lymphoma, Prognostic factor, R-CHOP

## Abstract

**Background:**

The objective of this study was to identify prognostic factors for survival in patients with primary diffuse large B-cell lymphoma (DLBCL) of the adrenal gland.

**Methods:**

Thirty one patients diagnosed with primary adrenal DLBCL from 14 Korean institutions and treated with R-CHOP (rituximab, cyclophosphamide, doxorubicin, vincristine and prednisone) were analyzed.

**Results:**

Complete remission (CR) and overall response rate after R-CHOP chemotherapy were 54.8% and 87.0%. The 2-year estimates of overall survival (OS) and progression-free survival (PFS) were 68.3% and 51.1%. In patients achieving CR, significant prolongations of OS (*P* = 0.029) and PFS (*P* = 0.005) were observed. Ann Arbor stage had no influence on OS. There was no significant difference in OS between patients with unilateral involvement of adrenal gland and those with bilateral involvement. When staging was modified to include bilateral adrenal involvement as one extranodal site, early stage (I or II) significantly correlated with longer OS (*P* = 0.021) and PFS (*P <*0.001).

**Conclusions:**

Contrary to prior reports, our data suggests that outcomes of primary adrenal DLBCL are encouraging using a regimen of R-CHOP, and that achieving CR after R-CHOP is predictive of survival. Likewise, our modified staging system may have prognostic value.

## Background

Primary extranodal non-Hodgkin’s lymphoma (NHL) is characterized by disease confined wholly or chiefly to an extranodal site [1]. The adrenal gland is an extremely rare site of primary extranodal NHL, accounting for less than 1% of all NHL and only 3% of primary extranodal lymphomas [2,3]. While bilateral involvement of adrenal gland, predominantly male and elderly, and frequent association with adrenal insufficiency are commonly reported clinical features of primary adrenal NHL, diffuse large B-cell lymphoma (DLBCL) is the most common histologic subtype, with a non-germinal center B-cell (non-GCB) phenotype [4-7]. To date, only about 120 publications (case reports or patient series) appear in the literature, each limited in scope. Conclusions have thus been drawn from clinical data and treatment outcomes of these reviews, which may not accurately reflect the nature of primary adrenal NHL.

The prognosis of primary adrenal DLBCL has heretofore been considered quite poor, entailing 1-year overall survival (OS) rate as low as 17.5% [3,8,9]. Because treatment modalities have varied widely, including surgical resection, radiation, and chemotherapy, optimal treatment of primary adrenal DLBCL lacks consensus. The patients with primary adrenal DLBCL had been treated with CHOP (cyclophosphamide, doxorubicin, vincristine and prednisone) like chemotherapy before rituximab era and central nerve system (CNS) relapse was frequent [10]. At present, it is unclear whether R (rituximab)-CHOP truly improve the treatment outcomes in these patients, although a few reports show that major improvement is possible [5,11]. Consequently, further studies with a larger number of patients are warranted.

The purpose of this retrospective, multicenter study was to evaluate the clinical characteristics and treatment outcomes of the patients with primary adrenal DLBCL, focusing on parameters predictive of survival in the patients with primary adrenal DLBCL who treated with R-CHOP.

## Results

### Baseline characteristics

Of our initial cohort, DLBCL was histologically confirmed in 86.7% (39/45), while one diagnosis of Burkitt’s lymphoma was rendered, and five other patients had T cell lymphomas such as anaplastic large cell lymphoma, peripheral T-cell lymphoma, not otherwise specified and extranodal NK/T cell lymphoma, nasal type. Because of the poor PS, 12.8% (5/39) of patients with DLBCL did not received chemotherapy. Another 7.6% (3/39) of patients were treated with a CHOP regimen, prior to the advent of rituximab. Ultimately, 79.4% (31/39) of patients with primary adrenal DLBCL (who received R-CHOP) were eligible for analysis. Among them, 93.7% (15/16) were non-GCB type by immunohistochemistry, and the median value of Ki-67 expression was 80% (range, 50%-98%) from the 19 tested patients.

The median age of patients was 64 years (range, 36–78 years), with the male to female ratio of 2.8:1. Magnitudes of adrenal masses ranged from 2.0-29.8 cm (8.4 cm, median). Sixteen patients (51.6%) presented with abdominal pain, 10 patients (32.2%) complained of weight loss, and 3 patients (7.7%) were diagnosed incidentally. B symptoms were observed in 15 patients (51.7%). At presentation, 19 patients (61.3%) had bilateral adrenal disease, 10 patients (32.2%) fulfilled criteria for bulky disease, and Eastern Cooperative Oncology Group (ECOG) performance status (PS) was poor (ECOG PS ≥2) in 4 patients (12.9%). Adrenal insufficiency was confirmed in 37.5% (6/16) of patients who were tested, and elevated serum lactic dehydrogenase (LDH) was found in 87.1% (27/31). Twenty-four patients (77.4%) had the number of extranodal involvement sites more than one. There was no bone marrow or CNS involvement of lymphoma at the time of diagnosis. Patients characteristics were described in Table 1.

**Table 1:** Patients characteristics

**Characteristics**	**Number of patients**	**Percentage**
Age		
≤ 60	12	38.7
> 60	19	61.3
Gender		
Male	23	74.2
Female	8	25.8
ECOG PS		
0-1	27	87.1
2-4	4	12.9
B symptom		
Absent	14	48.3
Present	15	51.7
Bulky disease		
Absent	21	67.7
Present	10	32.3
LDH		
Normal	4	12.9
Elevated	27	87.1
Primary site of adrenal gland		
Unilateral	12	38.7
Bilateral	19	61.3
Adrenal insufficiency		
Absent	10	62.5
Present	6	37.5
Ann Arbor stage		
IE, IIE	5	16.1
IIIE, IV	26	83.9
Number of extranodal sites		
0-1	7	22.6
2 or more	24	77.4
Ki-67 LI		
> 80%	11	57.9
≤ 80%	8	42.1
IPI		
Low/Low-intermediate	8	25.8
High-intermediate/High	23	74.2

ECOG PS, Eastern Cooperative Oncology Group performance status; LDH, lactate dehydrogenase; Ki-67 LI, Ki-67 labeling Index; IPI, International Prognostic Index.

### Treatment and outcomes

**Figure 1:**
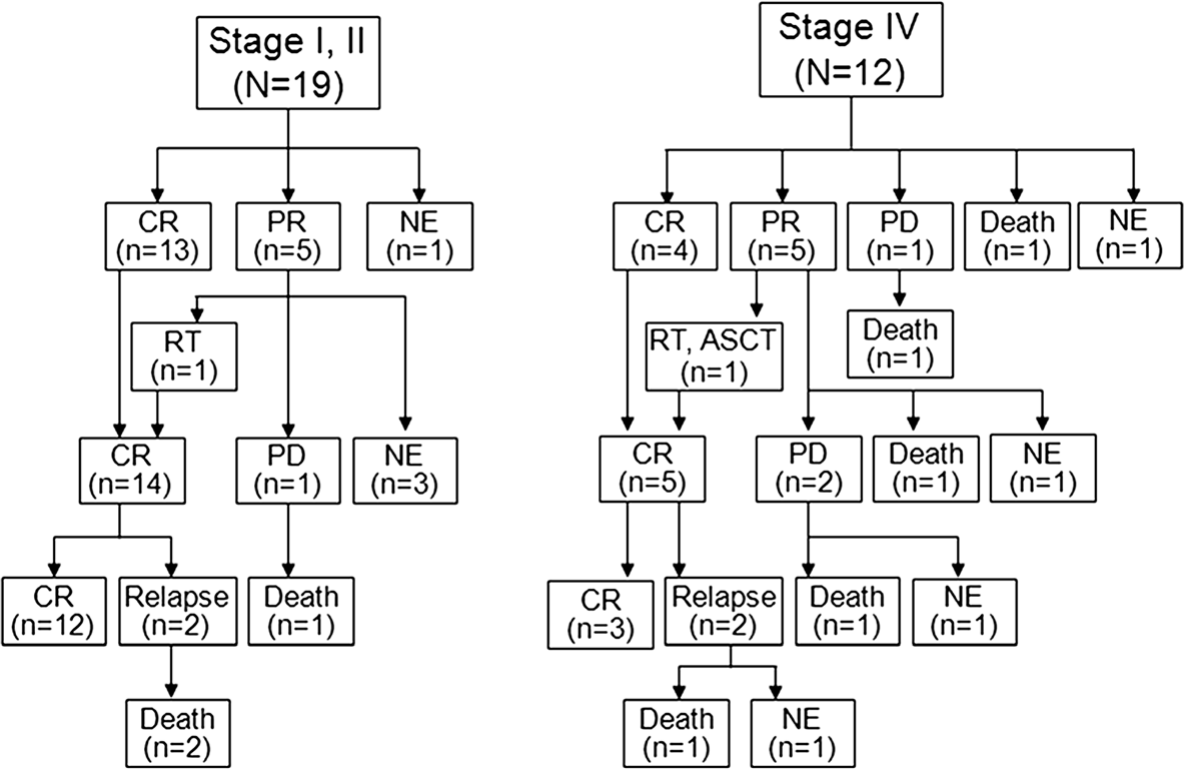
Management flow chart according to the modified stage; CR, complete remission; PR, partial remission; PD, progressive disease; NE, not evaluable; RT, radiotherapy; ASCT, autologous stem cell transplantation.

Seven patients (22.6%) received adrenalectomy for diagnostic and therapeutic purposes, prior to R-CHOP chemotherapy. However, adrenalectomy conferred no survival benefit (*P* = 0.979). The 31 primary adrenal DLBCL patients received median 6 cycles of R-CHOP chemotherapy (range, 1–8 cycles). Treatment response evaluated in 90.3% (28/31) of these patients showed complete remission (CR) in 54.8% (17/31) and partial remission (PR) in 32.3% (10/31). One patient (3.2%) developed progressive disease (PD). Of the 10 patients attaining PR with R-CHOP, 6 patients received additional therapy. Two patients achieved CR—one after adrenal irradiation; the other following autologous stem cell transplantation (ASCT) and radiotherapy—while 1 patient died due to treatment related mortality (TRM). Three patients developed disease progression despite salvage chemotherapy. The remaining 4 patients were lost to follow-up. Flow of treatment is shown in Figure 1.

**Figure 2:**
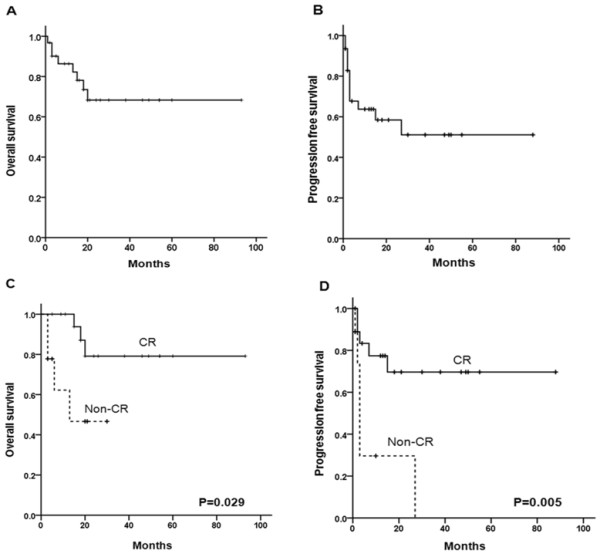
**Kaplan-Meier survival analysis of primary adrenal DLBCL treated with R-CHOP.** Overall survival (**A**) and progression-free survival (**B**) of 31 patients with primary adrenal DLBCL. Overall survival (**C**) and progression-free survival (**D**) according to the response of R-CHOP.

Median OS and PFS were not reached during a median follow-up duration of 18 months (range, 1–93 months). The 2-year estimates of OS and PFS were 68.3% and 51.1%, respectively (Figure 2A and B). Eight patients died in the follow-up period—one patient (3.2%) from TRM (pneumonia) during R-CHOP chemotherapy, 3 (9.6%) from PD, and 4 (12.9%) due to TRM (3 pneumonia, 1 Steven-Johnson Syndrome) during salvage chemotherapy. Significantly longer OS (*P* = 0.029, Figure 2C) and PFS (*P* = 0.005, Figure 2D) were observed in the patients achieved CR after R-CHOP chemotherapy compared to the patients who failed to achieve CR.

### Failure patterns of treatment

Among the 19 patients who achieved CR (17 after R-CHOP and 2 after R-CHOP followed by radiotherapy or ASCT), 4 patients (21.0%) suffered CNS (n = 2) or nodal (n = 2) relapse. Of all patients who relapsed, 2 TRM occurred during salvage chemotherapy. Successive CR was never observed in the patients treated with salvage therapy. Three of the initial 10 patients achieving PR showed disease progression after salvage chemotherapy, involving either CNS (n = 2) or adrenal gland (n = 1). None of the 4 patients with CNS relapse or progression had received intrathecal prophylaxis.

### Analysis of prognostic factors

By conventional Ann Arbor staging system, only 5 patients (16.1%) classified as early stage (stage IE or IIE), while the majority (26 patients, 83.9%) belonged to advanced stage (stage IIIE or IV). There was no survival difference between the patients with early stage and advanced stage (*P* = 0.445). None of the International Prognostic Index (IPI) criteria (age >60 years, *P* = 0.232; elevated LDH, *P* = 0.738; poor PS, *P* = 0.593; the number of extranodal involvement sites more than one, *P* = 0.979) impacted OS. According to IPI, 1 patient (3.2%) was considered low risk, 7 (22.6%) were low-intermediate, 11 (35.5%) were high-intermediate, and 12 (38.7%) were high risk. Altogether, 23 patients (74.2%) classified as high-intermediate or high risk patients. However, IPI stratification failed to equate with a OS difference (*P* = 0.142), as did Ki-67 expression >80% (*P* = 0.052), bulky disease (*P* = 0.647), presence of B symptoms (*P* = 0.841), and presence of adrenal insufficiency (*P* = 0.387).

**Figure 3:**
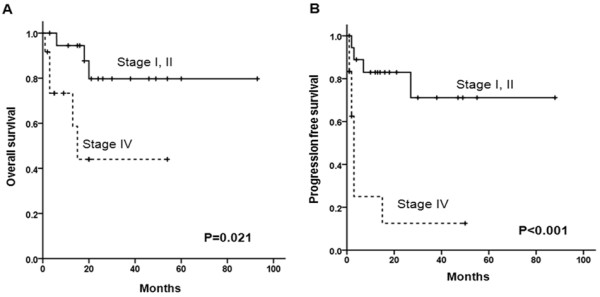
Overall surival (A) and progression-free survival (B) according to the modified stage in primary adrenal DLBCL.

As a consequence, we elected to modify primary adrenal DLBCL staging system as follows: 1) stage I was defined as the disease confined to the adrenal gland only, regardless of unilateral or bilateral involvement, 2) stage II included local or distant nodal disease of the abdomen, and 3) stage IV was indicative of disseminated extranodal disease or concomitant supra-diaphragmatic nodal involvement. With this modified staging system, 10 patients (32.3%) were stage I, 9 (29.0%) were stage II, and 12 (38.7%) were stage IV. Compared with advanced stage (stage IV), early stage (stage I or II) was significantly associated with better OS (*P* = 0.021, Figure 3A) and PFS (*P <*0.001, Figure 3B). Survival rates, however, were similar between stage I and stage II disease. Because bilateral involvement of adrenal gland did not significantly impact OS (*P* = 0.524) and PFS (*P* = 0.450), bilateral adrenal involvement was regarded as a single involvement of extranodal site in this modified staging system. The modified number of extranodal sites more than one thus corresponded with poor OS (*P* = 0.008) and PFS (*P* = 0.007). In multivariate analysis, our modified staging system showed significant survival difference for OS (*P* = 0.036, HR 4.73, 95% CI 1.10-20.4) and PFS (*P* = 0.003, HR 6.57, 95% CI 1.91-22.7).

**Figure 4:**
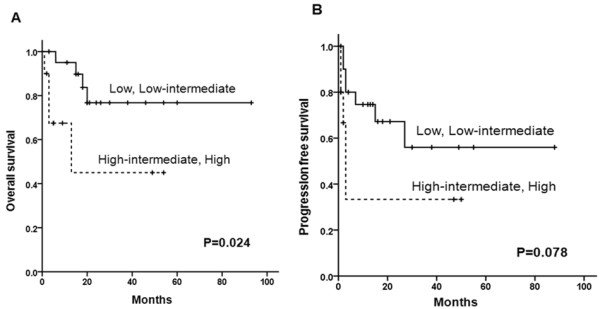
Overall surival (A) and progression-free survival (B) according to the modified IPI score using modified staging system.

IPI stratification was also revised as follows: 8 patients (25.8%) were low risk, 13 (41.9%) were low-intermediate, 6 (19.4%) were high-intermediate, and 4 (12.9%) were high risk. A total of 10 patients (32.2%) patients were subsequently assigned to high-intermediate or high risk according to the modified IPI. When these 4 risk categories were further grouped for survival comparison (low or low-intermediate *vs.* high-intermediate or high risk patients), OS showed a significant difference (*P* = 0.024, Figure 4A). There was only a trend of difference in PFS according to the modified IPI (*P* = 0.078, Figure 4B).

## Discussion

Compared with nodal DLBCL, this study has shown that primary adrenal DLBCL frequently accompanied by many adverse features such as bulky disease, elevated LDH, and advanced stage. Nevertheless, we have determined that therapeutic outcomes with R-CHOP are much better than previously reported [3,6,8]. Of particular import, OS of the patients with primary adrenal DLBCL is not affected by bilateral involvement of adrenal gland, Ann Arbor staging system and IPI score. Therefore, we implemented a modified staging system, similar to Lugano staging system for gastrointestinal (GI) lymphoma. With our approach, bilateral involvement of adrenal gland was considered as a single-site extranodal involvement, enabling more accurate prediction of survival (OS and PFS) in primary adrenal DLBCL.

The definition of primary extranodal NHL is a still controversial [12]. We defined that primary adrenal DLBCL be confirmed histologically and dominant adrenal gland mass regardless of regional lymph node involvement. And we also included the patients with both dominant adrenal gland mass and other extranodal involvement or concomitant supra-diaphragmatic nodal involvement in this study because the patients with DLBCL usually associated with aggressive clinical features [13]. In doing so, the size of adrenal mass was required to exceed 5 cm (reflecting TNM staging system of adrenal gland cancer) to exclude the DLBCL from other extranodal origin [14].

As with previous reports, the most common pathologic subtype of primary adrenal NHL was DLBCL (86.7%) and most (93.7%) were non-GCB phenotype in this study.

Optimal treatment of primary adrenal DLBCL is currently open to debate. In earlier trials of CHOP or CHOP-like therapy, OS of 20%-50% has been reported [6]. Our data revealed a CR rate of 54.8% and PR rate was 32.3% after R-CHOP therapy, with 2-year estimates of OS and PFS at 68.3% and 51.1%, respectively. According to the previous reports, advanced age, larger size of tumor, presence of adrenal insufficiency, bilateral involvement of adrenal gland, and elevated LDH were related with poor prognosis in primary adrenal NHL [7,15,16]. Most patients with primary adrenal DLBCL accompanied by a number of these adverse features. Most were elderly with bulky disease and/or elevated LDH. A non-GCB phenotype also predominated, as well as advanced stage. Nevertheless, our treatment outcomes with R-CHOP for primary adrenal DLBCL were not inferior to those of nodal DLBCL [17,18]. R-CHOP combination chemotherapy may thus be a reasonable first-line choice for treatment of primary adrenal DLBCL. It could overcome the effect of adrenal insufficiency. With CR after R-CHOP, OS and PFS were clearly prolonged in our analysis. Using modified staging system, however, only 33.3% (4/12) of patients with advanced stage primary adrenal DLBCL achieved CR after R-CHOP (Figure 1). Therefore, more aggressive treatment (ie, ASCT consolidation after R-CHOP) should be considered in the patients with advanced stage of primary adrenal DLBCL according to the modified staging system.

While a majority of patients (74.2%) were initially assigned high-intermediate or high risk IPI in this study, conventional IPI may not be valid in primary adrenal DLBCL for prognostic purposes. Most patients are >60 years old (61.3%) and have advanced disease (83.9%), with elevated LDH (87.1%) and multiple (2 or more) extranodal involvement (77.4%). In our analysis, bilateral adrenal involvement does not constitute advanced (stage IV) disease, because there was no OS difference between bilateral and unilateral adrenal gland involvement (*P* = 0.524). Furthermore, we found that with modification of the number of extranodal involvement (bilateral involvement of adrenal gland was considered as a single-site extranodal involvement), OS (*P* = 0.008) and PFS (*P* = 0.007) actually differ significantly for advanced *vs.* early stage of primary adrenal DLBCL. Accrual of involved extranodal sites for paired organs in Ann Arbor staging system still remains controversial. Bilateral involvement of primary breast DLBCL was classified as stage IV [19], while primary lymphoma of both testes classified as stage I [13]. Consequently, bilateral primary adrenal DLBCL also might be reasonable to be classified as stage I and single involvement of extranodal site.

Ann Arbor staging system has already been shown unsuitable for some types of extranodal NHL, namely GI lymphoma. In our study, survival differences according to the conventional Ann Arbor staging system went undetected, because most patients (77.4%) were stage IV by Ann Arbor staging system. We therefore instituted modifications similar to the Lugano staging system for GI lymphoma [20], defining 3 stages of primary adrenal DLBCL by extent of disease and nodal dissemination. Theoretically, there was no stage III disease in our new definition. With this proviso, early stage (stage I or II) disease fared significantly better in terms of OS and PFS, compared with advanced stage (stage IV) disease. Even with modified staging system, survival was comparable for stage I and II localized disease. On the other hand, modified IPI stratification resulted in a significant difference in OS for low or low-intermediate *vs.* high-intermediate or high risk IPI groups, while PFS did not differ. Because conventional IPI criteria yielded no survival differences, new modified IPI should be considered for evaluating the prognosis of primary adrenal DLBCL.

Recent study reported that the risk of CNS relapse was high in primary adrenal NHL [6,10]. While the incidence of CNS disease at diagnosis is likely lower than general estimates, our data showed CNS relapse in four patients (12.9%), while two suffered CNS relapse after R-CHOP therapy, and another two relapsed in CNS after salvage chemotherapy. The rate of CNS recurrence in aggressive NHL (without CNS prophylaxis) is on the order of 2.2%-5% [21-23]. Hence, CNS prophylaxis might be considered in primary adrenal DLBCL like other extranodal NHL such as primary testicular lymphoma.

Limitations of this study include its small patient population and retrospective nature. Unfortunately, a prospective, randomized trial would be difficult to conduct, given the exceptionally low disease incidence. Although another published case series (n = 15) of primary adrenal NHL has emerged from British Columbia [6], the present analysis is the largest known study of primary adrenal DLBCL thus far.

## Conclusions

Our findings suggest that R-CHOP combination chemotherapy is an effective first-line regimen for primary adrenal DLBCL, despite the inherently poor prognosis of this disease. Furthermore, our staging modifications, which redefine extranodal involvement, more accurately reflect treatment outcomes and may be preferential in this setting.

## Patients and methods

### Patient population

From 14 Korean institutions, a total of 45 patients with primary adrenal NHL, newly diagnosed between January, 1998 and December, 2011, were retrospectively reviewed. Thirty nine (86.7%) patients were diagnosed as primary adrenal DLBCL. Among them, 31 patients were treated with R-CHOP and reviewed in this study. Primary adrenal DLBCL was defined as follows: (1) histologic confirmation as DLBCL, with dominant adrenal gland mass regardless of regional lymph node involvement, and (2) absence of leukemic manifestations [24]. Patients with disease of other extranodal sites or lymph nodes above diaphragm were included in this analysis, provided their largest extranodal mass was adrenal-based and reached a relatively large size (> 5 cm) [25]. In DLBCL patients, molecular classification as GCB or non-GCB relied on Hans criteria [26]. The percentage of tumor cells with Ki-67 nuclear antigen expression was evaluated in immunophenotypic studies. Patient characteristics, as documented in medical records, were retrospectively examined to identify potential prognostic variables. These variables included age, gender, ECOG PS, initial presenting symptoms, presence of B symptoms, magnitude of adrenal gland mass, presence of adrenal insufficiency, pertinent laboratory determinants (ie, electrolytes, serum LDH), and related treatment data. Bulky disease was defined as a adrenal gland mass with the largest dimension greater than 10 cm. Adrenal insufficiency, when clinically suspected, was diagnosed via cortisol level or rapid ACTH stimulation test. None of the patients had taken immunosuppressive agents. This study was approved by the Institutional Review Board of each participating institution.

### Stage and prognostic index

All patients were staged in accordance with the Ann Arbor staging system [27], classifying unilateral primary adrenal DLBCL as stage IE and bilateral primary adrenal DLBCL as stage IV. Using IPI criteria (age >60 years, stage III/IV disease, elevated LDH level, ECOG PS ≥2, and more than one extranodal site of disease) [28], each patient was stratified to one of four risk groups by IPI: 0 to 1, low risk; 2, low-intermediate risk; 3, high-intermediate risk; and 4 to 5, high risk.

### Treatment and response

Treatment outcomes were analyzed in primary adrenal DLBCL patients who treated with R-CHOP. The R-CHOP regimen was administered by intravenous (IV) or oral (PO) route in 21-day cycles: rituximab (375 mg/m^2^, IV), cyclophosphamide (750 mg/m^2^, IV), doxorubicin (50 mg/m^2^, IV), vincristine (1.4 mg/m^2^, IV) on Day 1 and prednisone (100 mg, PO) on Days 1–5. Cerebrospinal fluid was examined only if clinically indicated. Response was assessed after completion of the R-CHOP chemotherapy, using International Working Group criteria [29].

### Statistical analysis

OS was measured from the first date of diagnosis until death from any cause, with surviving patients were censored at the last follow up date. PFS was defined from the first date of diagnosis until disease progression, relapse after response or death due to lymphoma or treatment related. Other causes of death or surviving patients at last follow up were censored. Survival curves were plotted by the Kaplan-Meier method and compared using the log-rank test. The influence of each prognostic factors identified by univariate analysis was assessed by multivariate analysis using Cox’s proportional-hazards regression by stepwise method. *P*-value <0.05 was considered statistically significant in all analyses. All statistical analyses were performed using the SPSS for Windows, Version 18.0.

## Abbreviations

DLBCL, Diffuse large B-cell lymphoma; CR, Complete remission; R-CHOP, Rituximab- cyclophosphamide, doxorubicin, vincristine and prednisone; OS, Overall survival; PFS, Progression-free survival; NHL, Non-Hodgkin’s lymphoma; Non-GCB, Non-germinal center B-cell; CNS, Central nerve system; PS, Performance status; ECOG, Eastern Cooperative Oncology Group; LDH, Serum lactic dehydrogenase; PR, Partial remission; PD, Progressive disease; ASCT, Autologous stem cell transplantation; TRM, Treatment related mortality; IPI, International Prognostic Index; GI, Gastrointestinal.

## Competing interests

Non-financial competing interests

## Authors’ contributions

YRK involved in conception, design, data interpretation, and manuscript writing. JSK performed data interpretation and revising it critically for intellectual content. YHM, DHY, HS, YM involved in revising manuscript critically for important intellectual content. YP, YD, SHJ, JSP, SYO, SL involved in acquisition of data, analysis of data. EKP, JSJ, WL, HL, HE, SA involved in acquisition of data, analysis of data and participating in comprehensive discussion. JJ, SKB, SJK involved in analysis of data and interpretation of data. WSK, CS involved in analysis of data and participating in comprehensive discussion. All authors read and approved the final manuscript.
